# 2366. Profile and Outcomes of Vaccinated Adult Individuals with COVID-19 Breakthrough Infections Hospitalized in a Tertiary Hospital in the Philippines

**DOI:** 10.1093/ofid/ofad500.1987

**Published:** 2023-11-27

**Authors:** Mark Ramon Victor B Llanes, Regina Berba

**Affiliations:** Southern Philippines Medical Center, Davao City, Davao del Sur, Philippines; University of the Philippines Manila, Manila, National Capital Region, Philippines

## Abstract

**Background:**

Characterization of coronavirus disease 2019 (COVID-19) vaccine breakthrough infections are limited. This study aims to characterize breakthrough infection hospitalized in a tertiary hospital in the Philippines and identify risk factors associated with severe and critical outcomes.

**Methods:**

A retrospective cohort study was done on 133 vaccinated adult individuals with COVID-19 breakthrough infection hospitalized in a tertiary hospital since the start of the vaccination roll out in the Philippines. Demographic, clinical and vaccination profile were obtained and statistically correlated with outcome of severe and critical COVID-19 illness.

Work Flow Diagram

**Figure:**
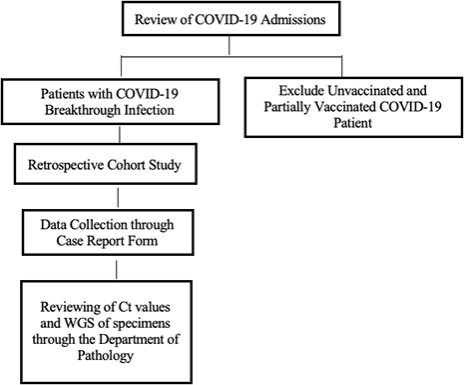

Work Flow Diagram of Determining COVID-19 Breakthrough Infections

**Results:**

A total of 133 patients were reviewed: the mean age was 53 years. The mean time to SARS CoV2 detection from full dose vaccination is 107 days. Most of the patients have hypertension (n=76), diabetes mellitus (n=49) and chronic kidney disease (n=30). Majority have moderate severity (n=56). Age of more than 65 (OR=1.358, P=0.045), presence of acute coronary syndrome (OR=17.869,95%, P=0.048), active cancer (OR=6.103, P=0.006), and diabetes mellitus (OR=2.652, P=0.031), symptomatology of cough (OR=3.538, P=0.024), shortness of breath (OR=3.362, P=0.019), and fatigue (OR=3.11, P=0.029) and a high baseline sensitivity C-reactive protein (OR=1.006, P=0.017) were associated with increased risk of developing severe and critical COVID-19 breakthrough infection. On the other hand, receipt of Pfizer as primary series vaccination (OR=0.190, P=0.032) and a high baseline PaO2/FiO2 ratio (OR=0.993, P< 0.01) were associated with decreased risk of developing severe and critical illness.

Demographic and Clinical Profile (Categorical Variables) of patients with COVID-19 Breakthrough Infections hospitalized in Philippine General Hospital

**Figure:**
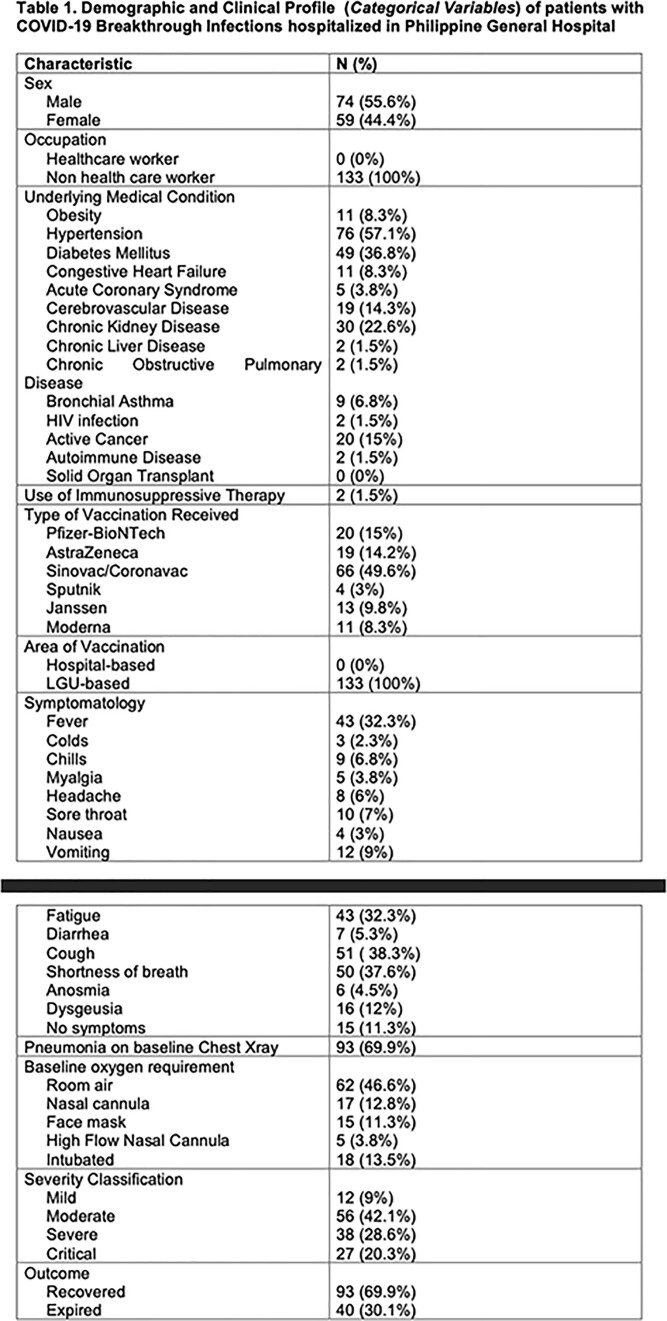

Demographic and Clinical Profile (Categorical Variables) of patients with COVID-19 Breakthrough Infections hospitalized in Philippine General Hospital

Demographic and Clinical Profile (Continuous Variables) of patients with COVID-19 Breakthrough Infections hospitalized in Philippine General Hospital

**Figure:**
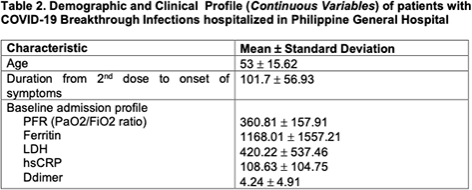

Demographic and Clinical Profile (Continuous Variables) of patients with COVID-19 Breakthrough Infections hospitalized in Philippine General Hospital

Laboratory Profile (Cycle Threshold) of COVID-19 Breakthrough Infection Hospitalized in Philippine General Hospital

**Figure:**
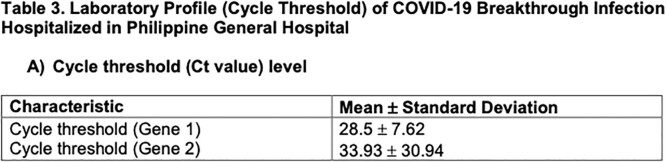

Laboratory Profile (Cycle Threshold) of COVID-19 Breakthrough Infection Hospitalized in Philippine General Hospital

**Conclusion:**

Majority of the COVID-19 breakthrough infections have moderate disease. Age more than 65 years old, presence of co-morbidities such as acute coronary syndrome, active cancer and diabetes mellitus, symptoms of cough, shortness of breath and fatigue on initial presentation and high baseline high sensitivity CRP were associated with increase chance of developing severe and critical COVID-19 among breakthrough patients, while a primary series vaccination with Pfizer and a high baseline PaO2/FiO2 ratio were associated with decreased risk.

Association of Demographic and Clinical Profile and Laboratory Profile of COVID-19 Breakthrough Infection in developing Severe and Critical COVID-19

**Figure:**
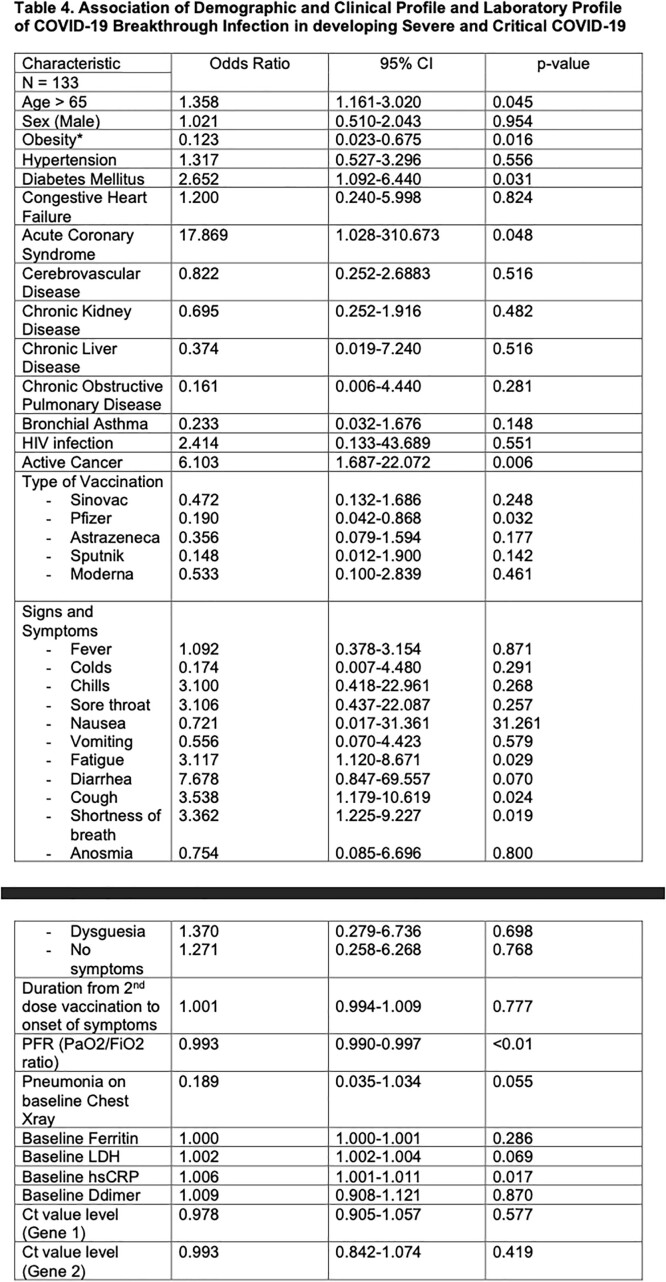

Association of Demographic and Clinical Profile and Laboratory Profile of COVID-19 Breakthrough Infection in developing Severe and Critical COVID-19

**Disclosures:**

**All Authors**: No reported disclosures

